# Case Method in COPD education for primary care physicians: study protocol for a cluster randomised controlled trial

**DOI:** 10.1186/s13063-017-1889-4

**Published:** 2017-04-27

**Authors:** Hanna Sandelowsky, Ingvar Krakau, Sonja Modin, Björn Ställberg, Anna Nager

**Affiliations:** 1 Division of Family Medicine and Primary Care, NVS, Karolinska Institutet, Alfred Nobels Allé 23, D2, Huddinge, Stockholm 14183 Sweden; 2 Department of Public Health and Caring Science, Family Medicine and Preventive Medicine, Uppsala University, Uppsala, Sweden

**Keywords:** Case methodology, Continuous medical education, Professional training, Primary care physicians, COPD, Primary care, Cluster randomised controlled trial

## Abstract

**Background:**

Chronic obstructive pulmonary disease (COPD) is a common cause of morbidity and mortality worldwide. It is often undiagnosed and insufficiently managed. Effective forms of continuing medical education (CME) for primary care physicians (PCPs) are necessary to ensure the implementation of guidelines in clinical practice and, thus, improve patients’ health.

**Methods:**

In this study, we will measure the effects of CME by Case Method and compare them against those of traditional lectures and no CME at all through an unblinded, cluster randomised controlled trial (CRCT). Thirty-three primary health care centres (PHCCs) in Stockholm, Sweden, with a total of 180 PCPs will be involved. Twenty-two primary PHCCs, will be cluster-randomised into: an intervention group who will receive CME by Case Method (*n* = 11) and a control group who will receive traditional lectures (*n* = 11). The remaining PHCCs (*n* = 11) will be a reference group and will receive no CME. From the intervention and control groups, 460 randomly selected patients with COPD in GOLD stages 2 and 3 will participate, while no patients will be recruited from the reference group.

For the patients, smoking status, actual treatment and urgent visits to a health provider due to airway problems will be registered. For the PCPs, professional competence (i.e. knowledge and management skills) in COPD, will be measured using a questionnaire based on current guidelines and guideline implementation problems in clinical practice which has previously been described by the authors. Data will be collected at baseline and at follow-up, which will be after 1.5 years for the patients, and 1 year for the PCPs.

Statistical methods for individual-level and cluster-level analyses will be used.

**Discussion:**

COPD is considered a particularly complex clinical challenge involving managing multimorbidity, symptom adaptation, and lifestyle problematisation. Case Method in CME for PCPs may contribute to a better understanding of the impact of COPD on patients’ lives and, thus, improve their management of it. The present study is expected to contribute scientific knowledge about indicators for an effective CME in COPD that is tailor-made to primary care physicians.

**Trial registration:**

ClinicalTrials.gov, identifier: NCT02213809. Registered on 10 August 2014.

Protocol version: Issue date: May 2014.

**Electronic supplementary material:**

The online version of this article (doi:10.1186/s13063-017-1889-4) contains supplementary material, which is available to authorized users.

## Background

In recent years, the management of chronic obstructive pulmonary disease (COPD) has improved and awareness of the importance of an early detection and treatment of COPD among patients and primary care providers has increased [1]. In spite of this, under-diagnosis and late detection continue to occur and current guidelines are still poorly adhered to [1–4]. To narrow the gap between theory and practice, more studies on the implementation of COPD guidelines in primary care practice are needed [5].

The skills of primary care physicians (PCP) in detecting and managing COPD are of great importance for patients [6]. Continuing medical education (CME), tailored to the needs of primary care, may be an important tool to improve guideline adherence and implementation. The effectiveness of CME is largely dependent on how well suited the design and content of the educational programme are to the target group. Effectiveness can be measured by way of three aspects: competence, performance, and patient health status [7]. A decline in effectiveness with regard to these three aspects has been previously shown and it has been deemed adequate at best [8]. However, educational outreach visits appear to improve care [9], are feasible at primary care settings and are well received by care professionals when heavy workload and time constraints often lead to poor attendance at CME sessions outside the workplace (Berggren, E. In manuscript). A didactic educational style involving lectures and textbook instruction instead of hands-on training, have been the norm even in advanced training for physicians. However, in the last few decades, interactive CMEs have become more common. Today, it is widely accepted that a combined approach involving both interactive and didactic forms of education is more effective than either of these on their own [10].

Case Method can be used to create an interactive type of CME for a particular area of interest or a specific problem based on the professionals’ perspective. [11]. According to an earlier Swedish study, use of case-based training for implementing evidence-based practice in primary care was associated with decreased mortality in patients with coronary heart disease [12]. In addition, an American study showed a 50% increase in evidence-based treatment and management of COPD among PCPs who received case-based CME compared with colleagues who did not [13]. However, the effectiveness of interactive CME in COPD carried out at medical centres, or primary health care centres (PHCCs), with both physicians and patients as endpoints, has not been previously studied. We believe that a cluster randomised trial that uses the PHCC as a randomisation and analysis unit would be a suitable study design as it takes into account the effect of CME reaching all PCP staff of a PHCC at once.

### Objective and hypothesis

The overall objectives of this randomised controlled trial pertain to individual patients and PCPs. The effectiveness of Case Method in COPD CME for PCPs will be evaluated and compared to that of traditional lectures as well as lack thereof. The main aim is to evaluate and compare the effects of CME on physicians’ competence with regard to the COPD care that patients will receive. The primary outcome measure is the difference in the mean total score on *The Clinical COPD Questionnaire* (CCQ) [14], a validated tool to evaluate disease-specific health status in patients with COPD. Secondary outcome measures include the difference in the total score on *The COPD Assessment Test* (CAT), another validated disease-specific questionnaire measuring health status [15] and in *The Lung Information Needs Questionnaire* (LINQ), which measures patient’s satisfaction in COPD care and information received [16]. At the same time, changes in COPD medication and participation in pulmonary rehabilitation, number of exacerbations, and smoking habits will be recorded.

Apart from evaluating effects, a description of the participating PHCCs will be performed focussing on the organisation of COPD care that they offer to their patients; for example, whether they have a nurse-led COPD clinic in the PHCC or not.

Our hypothesis is that CME for PCPs that is based on Case Method is more effective than traditional lectures or no CME at all in improving the disease-specific health status of COPD patients, due to physicians’ improved knowledge and skills in COPD management.

## Methods and design

This paper was written in line with the SPIRIT (Standard Protocol Items: Recommendations for Interventional Trials) 2013 explanation and elaboration: guidance for protocols of clinical trials [17], and the CONSORT (Consolidated Standards of Reporting Trials) 2010 Statement: extension to cluster randomised trials [18]. The SPIRIT Checklist and flow chart were used, see Additional file 1 and Fig. 2.

### Study design

**Fig. 1:**
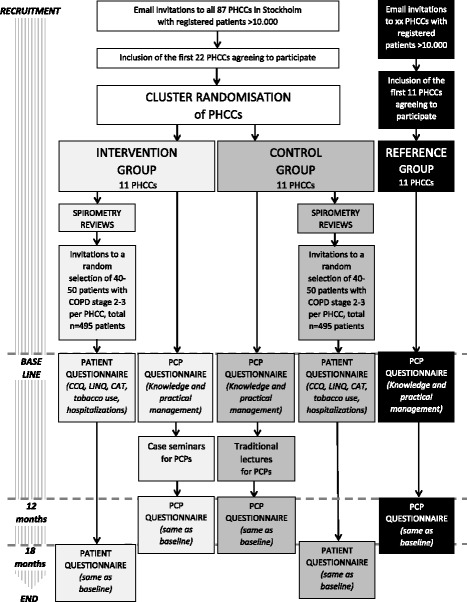
Flow chart of the trial

**Fig. 2:**
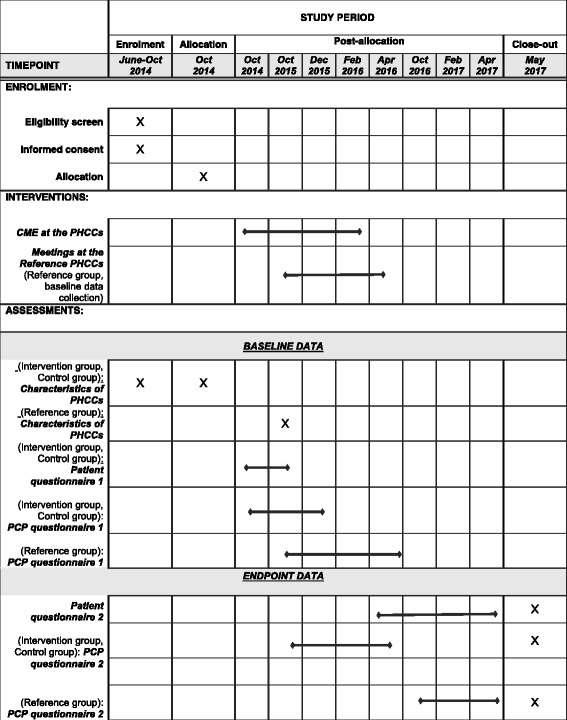
Enrolment, interventions and assessments according to Standard Protocol Items: Recommendations for Interventional Trials (SPIRIT)

This study involves a pragmatic and unblinded cluster randomised controlled trial (CRCT) with PHCCs as units of randomisation. In order to isolate the effect of CME, by Case Method, three groups (arms) will be established: one where CME by Case Method will be offered (arm 1, 11 PHCCs), one where traditional lectures (arm 2, 11 PHCCs) will be offered, and one where no CME will be offered at all (arm 3, 11 PHCCs). There will be a total of 33 PHCCs, or 33 clusters, from Stockholm County. From these, 180 PCPs (60 PCPs for each arm) and 460 patients (230 for arms 1 and 2, no patients for arm 3) will be asked to participate. Each PHCC will provide 25–30 patients, which will yield the minimum of 460 patients with COPD in GOLD stages 2 or 3 [2]. The outcome measures will be recorded at baseline and at follow-up which will be after 1 year for PCPs and 1.5 years for patients. The trial design is summarised in Fig. 1 and Fig. 2 as flow charts.

### Participants

#### Eligibility

The study will include 33 PHCCs (clusters) in Stockholm County with more than 10,000 registered patients in each PHCC. Patients with COPD at stages 2 or 3 according to the GOLD criteria [2] who will be willing to participate will be included in the trial. Within each cluster, the participating PCPs will have acquired specialist training in family medicine with a minimum of 5 years of completed or ongoing training, according to the requirements by the National Board of Health and Welfare in Sweden http://www.socialstyrelsen.se/english/Documents/Doctors-specialist-medical-training-National-Board-of-Health-and-Welfare.pdf. To minimise the dropout risk due to short-term employments, temporary PCPs working or training at a PHCC will be excluded, though, by attending CME sessions, they may have an impact on study outcomes.

#### Recruitment of PHCCs and participants

##### Recruitment of the PHCCs

In Sweden, almost all PCPs are employed by PHCCs, which are medical centres run by county councils directly or by contracted private companies. According to the policies of county councils, all PHCCs must provide the general population with certain primary care services that are carried out by PCPs and district nurses. In practice, all COPD patients in GOLD stages 2 and 3, and many in stage 4, are identified, examined and managed entirely in primary care. Primary care rehabilitation units in Stockholm County are often independently organised and managed separately from PHCCs.

Managers and PCPs of all 80 medium-sized or larger PHCCs (defined as caring for more than 10,000 registered patients, typically yielding five to ten eligible physicians per PHCC), which represent 40% of all 205 PHCCs in Stockholm County, will be contacted by a letter (by post and e-mail) which will serve as an invitation to participate in the study. To meet the required number of patients determined by the power calculation, a minimum of 11 PHCCs per arm will be needed. The number of PHCC depends on access to spirometry results, since a spirometry-verified COPD diagnosis is needed for inclusion. In Stockholm County primary care, there are currently technical and patient confidentiality issues that prevent the collection of data from digitally shared medical records, thus limiting access to spirometry results. Socioeconomic and demographic factors as well as characteristics of the PHCC, such as staffing and access to a nurse-based asthma/COPD clinic within the PHCC, will be taken into consideration during the data analysis and appropriate adjustments will be made for these factors in the models. Signed Informed Consent Forms will be obtained from all PCPs and managers who agree to participate. The first 22 PHCCs signing up will be included in the study and randomised into one of the two interventions arms. The remaining PHCCs with more than 10,000 registered patients will be contacted and the first 11 that accept participation will form a reference group. These PHCCs will not receive a CME, which makes randomisation into a reference arm difficult to motivate ethically.

##### Recruitment of PCPs and data collection

PCPs will be recruited for all three study arms. After agreeing to participate, the manager of the PHCC will receive oral and written information about the study during a face-to-face meeting with the research project manager. The manager of the PHCC then will inform the PCPs that participation in the study will be optional. The PCPs who attend the CME sessions will then, again, be contacted and informed by the CME leader prior to collecting baseline data at the beginning of the first CME session. The reference group will be informed by their manager and by a researcher who will collect baseline data at a specific occasion at each PHCC. By taking the expected response (90%) and dropout rate (20%) into account at the end, the 33 PHCCs will be expected to engage approximately 180 eligible PCPs (60 per arm) whose signed Informed Consent Forms will be required. The PCPs will be informed of their right to withdraw from the study at any time without negative consequences.

The completed questionnaires will be collected by the research group and be put in an envelope and stored in a secure place. The endline data will be collected 1 year after a completed CME during a follow-up visit at each PHCC. The persons who will be absent will be contacted separately to collect their individual endline data.

##### Recruitment of patients and data collection

Patients will be recruited only for arms 1 and 2, i.e. the intervention and control groups. A total of 990 randomly selected patients with COPD in GOLD stages 2 and 3 [2] (495 in each arm, 40–50 randomly selected eligible patients per PHCC unit) will be invited to participate. With an expected response rate of 60% at baseline and 80% at endline, a total of 230 patients per arm will be included. To ensure the eligibility of the patients, a spirometry slip together with an *International Classification of Diseases, version 10* (ICD-10) diagnosis of COPD (J44.0–J44.9) in the PHCC’s medical records, will be included and reviewed by the research group. Patients with a diagnosis of COPD in the records, but who do not fulfil the criterion of a FEV_1_/FVC ratio <0.7, will be excluded. An independent person, such as a nurse or an administrator, will handle the patient codes for maintaining their anonymity as well as send out the invitation letters. Together with the invitation letter, the patients will receive information about the study, an Informed Consent Form to fill in and the questionnaire (Additional file 2) in a self-addressed envelope to return to the researchers. Two reminder letters will be sent to the patients who have not replied at 2 weeks apart. The patients will be informed that they can withdraw from the study at any time without negative consequences. After receiving the informed consent from the patient, the coding key will be handed over to the researchers. The patient recruitment period will last for 2 months prior to the CME intervention at each PHCC. The endline data will be collected by mail (including two reminders) 1.5 years after the PCPs have completed the CME.

Due to the relatively small-scale collection of data, a Data Monitoring Committee is not deemed necessary.

### Interventions

#### The CME programmes

The CME sessions will take place at the PHCCs. The five CME leaders with strong competence in COPD management and experience/training in Case Method when required, will run two 120-min sessions at each PHCC, at a maximum of 3 months apart. Each PHCC will be assigned the same CME leader, but only one of these two approaches will be used. Each leader will abide by the well-defined intended learning outcomes (ILOs) and contents of the CME (Table 1) but will be allowed to use different presentation materials, such as slide shows and handouts.

**Table 1:**
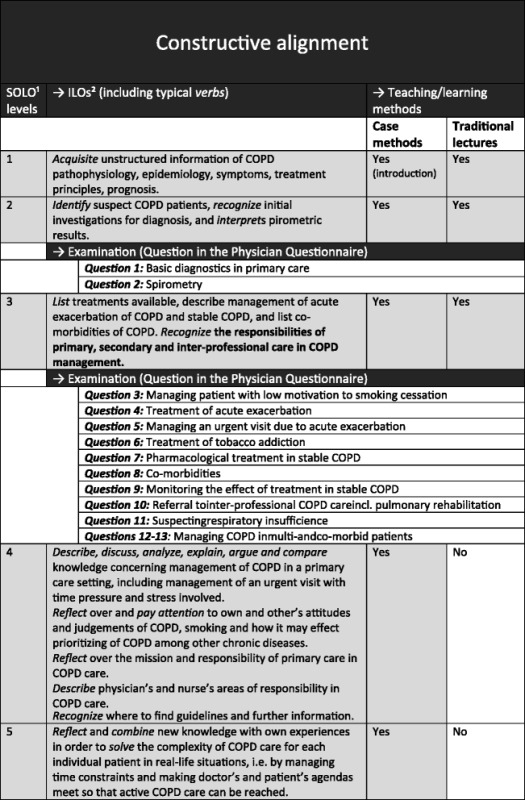
A summary of how the Constructive Alignment links the SOLO^a^ levels, ILOs^b^, teaching/learning methods and examination questions

^a^SOLO = Structure of Observed Learning Outcome taxonomy

^b^ILOs = intended learning outcomes

#### Patient-doctor consultations – care as usual

No interventions for patients will take place. Patients will visit their PHCC as usual during the study time.

#### Relevant parallel, permitted interventions

As new guidelines for COPD care in Sweden are expected to be published during the study period, the participating PCPs will not be asked to decline participation in other eventual COPD educational occasions during the study period. In any case, the PCPs will be likely recipients of information about updated COPD guidelines through different channels.

#### Description of CME by Case Method (intervention group, arm1)

After an initial 20-min introduction to the topic via a traditional lecture, the CME leader will start presenting a case seminar. The reason behind using cases lies in having students acquaint themselves with realistic problems or ‘cases’ with authentic, open-ended narratives out of realistic situations [19]. The cases will be read discussed at a seminar at each PHCC with 5–15 eligible PCPs. The two cases – one for each seminar – are described in Additional files 3 and 4. The emerging questions in a case have typically no obvious right or wrong answer – instead they can be answered in several acceptable ways. The CME leader will act more like a facilitator rather than an expert, encouraging the participants to try and solve problems through detailed discussions, reflection and collaboration, based on the knowledge, skills, and experiences that they already possess. Thus, the CME leaders will not always have clear-cut answers to possible questions from the participants – instead the questions will be up to the participants to answer. The primary aim is to improve decision-making skills in clinical practice. The learning outcome of a case seminar will largely depend on the participants’ level of activity and the facilitator’s ability to encourage the discussion in an open, respectful, and creative educational setting.

#### Description of sessions using traditional lectures (control group, arm 2)

Traditional lectures are lectures delivered in a didactic style, with the CME leader acting as an academic expert, i.e. they will decide on the content of the session and will teach by mainly one-way communication using slide show presentations as a pedagogical tool. Some interaction between the leaders and the students will occur, such as answering questions put forward by the students.

#### Intended learning outcomes (ILOs) of the CME

When designing the CME sessions, we will follow the Bologna Declaration [20]. We will start with the intended learning outcomes (ILOSs), and will then adjust teaching style and assessment method to fit those learning outcomes. This principle is an example of an outcome-based education and it is known as Bigg’s Constructive Alignment [21]. It is an influential educational theory that merges constructivism with alignment, i.e. the idea that ILOs, teaching and learning activities, assessments, and examination questions should work together (be aligned) to enable learners to achieve deeper levels of knowledge. Bigg’s Structure of Observed Learning Outcomes (SOLO) taxonomy [22] helps to map levels of understanding that can be built into the ILOs. SOLO levels 1–3 (S1 = prestructural, S2 = unistructural, S3 = multistructural) are typically integrated into the PCPs’ basic education, i.e. medical school. Levels 4–5 (S4 = relational, S5 = extended abstract) involve gradually increasing demands on reflection, hypothesising, creativity, discussion, and abstraction so that the learners can apply the new skills to new and broader areas [21, 23].

A summary of how the Constructive Alignment links the ILOs, teaching/learning methods and examination questions in our CME in COPD is shown in Table 1.

The intervention group (arm 1, CME by Case Method) will receive CME at SOLO levels 1–5 (S1–S5), with more focus on levels S3–S5, whereas the control group (arm 2, traditional lectures) will receive CME only at S1–S3. The contents of the ILOs are based on current evidence-based COPD guidelines [2, 24–26] and problem areas in managing COPD, as described in a previous study by the authors [27]. After analysing interviews with PCPs, the aforementioned study revealed problem areas from real-life primary care settings closely connected to time constraints during patient-doctor consultations that often lead to deprioritising of COPD. The examination questions will be based on ILOs emerging from S1–S3, hence covering both groups.

### Outcome parameters and planned statistical methods

Outcomes are chosen for their ability to assess the effect of the interventions on both patient and PCP individually as well as on PHCCs as a whole (cluster). For data collection, validated questionnaires together with study-specific questions will be used. The baseline data collection will be completed prior the first CME session for the PCPs. Endline data for PCPs and patients will be collected 1 year and 1.5 years, respectively, after a completed CME.

The primary outcome of the study will be the effect of CME on patients’ disease-specific health status. Hence, the power calculations for the study have been performed on the basis of the primary outcome measure for patients rather than PCPs.

#### Primary outcome measures for patients

A patient questionnaire (Additional file 2) consisting of three validated self-report instruments and study-specific questions will be used. The primary outcome measure will be the mean total score in *The Clinical COPD Questionnaire* (CCQ) which is a validated, self-administered questionnaire specifically developed to measure clinical control in patients with COPD [14]. It consists of 10 items, divided into three domains: symptoms, functional state, and mental state (scale per each item: 0 = best, 6 = worst). The higher the score, the greater the negative impact on quality of life and health status. The final measure is the mean value of the 10 questions; Minimal Clinically Important Difference = 0.44 of the final score (mean value) [28]. We will adjust for CCQ at baseline and the CCQs with the minimum of 6 of 10 items replied will be included in the analysis. The CCQ is constructed as an ordinal scale, and the statistics will be analysed accordingly. However, since the CCQ, as most symptom scales, is practically an interval scale, we will present the results using the mean instead of the median, to be able to compare our observations with those of earlier studies [29, 30].

#### Secondary outcome measures for patients

*The COPD Assessment Test (*CAT) will be used to access the impact of COPD on health status [15]. It consists of eight items covering coughing, phlegm in chest, chest tightness, breathlessness on exertion, limitations to doing activities at home, being confident leaving home, ability to sleep soundly, and feeling energetic. The scoring scale ranges between 0 and 5 for each item, and the total score ranges from 0 to 40. The higher the score, the greater the impact of COPD is on the patient’s health; Minimal Clinically Important Difference = 2 [31].

*The Lung Information Needs Questionnaire* (LINQ) will be used to assess the patient’s need for education and support in self-managing COPD, and their level of satisfaction with received COPD care. The questionnaire consists of 20 items that are divided into six categories: disease knowledge, medicine, self-management, smoking, exercise and diet [16]. The scoring scale ranges from 0 to 1, 0 to 2 or 0 to 3 for each item, and the total score ranges from 0 to 25. The higher the score, the greater the learning need; Minimal Clinically Important Difference = 1 [32].

Additional self-reported information on age, weight, length, educational level, tobacco use, and hospitalisations due to airway problems will be collected.

#### Outcome measures for PCPs

The outcome measures for the PCPs pertain to individual participants. The Physician Questionnaire (Additional file 5) has been constructed by the researchers. The questionnaire is in fact an outright examination in line with the Constructive Alignment: the items cover the ILOs of S1–S3, leaving out the ILOs of S4–S5, as the latter ones will only be applicable for the intervention group, as shown in Table 1. The questionnaire is designed as an assessment test consisting of five short patient cases, with two to three questions per case (13 in total). The questions touch upon ‘knowledge/skills’ and ‘practical management’ yielding 0–2 points each, and appear as either:

1. *Multiple choice questions.* These questions mainly cover the areas of ‘knowledge/skills’, based on the ILOs concerning the current guidelines for COPD care. For instance, interpreting a spirometry result will be tested by a multiple choice question, implying one correct answer out of seven options. However, for questions such as on correct treatment of COPD exacerbation, several options can be ticked. A correct answer gives 2 points, partially correct 1 point, and all other replies result in 0 points
2. *Open Questions.* These questions are based on the ILOs related to specific realistic problems in COPD management encountered in primary care, which have been described in a previous study by the author that was based on interviews with PCPs [27]. These problems are often faced in everyday practice and can be caused by deprioritising of COPD due to time pressure during a doctor-patient consultation. Open questions address issues such as ‘not becoming concerned due to clinical features’, ‘insufficient local routines for COPD care’, ‘negative personal attitudes and views about COPD’, ‘not managing COPD due to multimorbidity,’ and ‘perceiving a patient’s motivation as low’. For instance, two open questions address PCPs’ support of patients who try to quit smoking and provide PCPs with a possibility to state personal opinions and values or to describe own COPD management routines. Notably, each reply is considered correct only when it is in line with current COPD care guidelines, regardless of personal views on the subject. The free text replies will be analysed by quantitative content analysis, yielding 0–2 points according to a correction template, which will be constructed in alignment with the guidelines.
  Also, at baseline, PCPs’ characteristics, such as sex, age, and years in the profession, will be registered for use in the analysis and descriptions of responders and nonresponders.

#### Background factors at cluster level, for the PHCCs

The background factors for the PHCCs, such as patient demographics, number of patients per PCP, and details of a nurse-led COPD clinic within the PHCC, if applicable, will be registered at baseline and analysed at cluster level.

#### Data storage and security

The research group, i.e. the authors of this paper, will have access to the final trial dataset, and there are no contractual agreements that limit such access for the researchers.

### Sample size

#### Patient sample determines the number of PHCCs and PCPs

The design and execution of the study depend to a great extend on the power calculation of patient sample size, which, in turn, determines the PCP sample size. The power calculation is based on the Minimal Clinically Important Difference in average and standard deviation of 0.44 in the CCQ [14, 28]. According to power calculations, 230 patients in each intervention arm will be required (see ‘Recruitment’ above). Numbers of clusters will be 11 per arm, based on the sizes of the PHCCs. Unequal cluster sizes (5–10 PCPs) are expected due to variations in staff numbers at baseline and dropouts at endline. The coefficient of intracluster correlation (ICC) is set to 0.01 based on earlier studies on cluster randomisations in primary care [33, 34], a current cluster randomised educational intervention study among PCPs in Stockholm (Schmidt-Mende, K. In manuscript), and after recommendations from a statistician. Indication of uncertainty is based on the statistical variance between the clusters [35].

### Randomisation

#### Sequence generation and allocation procedure

The random assignment of the two active intervention groups will be performed by the members of the research group using a computer randomisation programme (http://www.random.org). Our study will not involve any form of restricted randomisation, such as matching or stratification. This is mainly due to the assumption that the participating PHCCs, all from Stockholm County, are fairly homogenous when it comes to size, staff and demographics. Also, earlier research [36] indicate that primary care settings similar to ours tend to possess a fairly low matching correlation (M-rho), hence barely meeting the previously established criterion for significance [37].

To make sure that there is no bias in the group allocation of participants, PHCCs will be selected first before randomisation of clusters takes place. This will be performed in two stages: the first after 3 months of recruitment, and the second after an additional recruitment period, if necessary, for reaching an appropriate sample size. Afterwards, both the research group and the participating PHCCs will be notified about which arm each PHCC will belong to. However, PCPs will sign their informed consent at the first CME session, thus after randomisation is complete. The patients who agree to participate and have signed Informed Consent Forms will fall under the cluster of their PHCCs, but will only be informed about their physicians receiving a CME in COPD and not about which study arm they will be part of.

### Statistical methods

The cluster randomised design provides protection against contamination across trial groups when trial patients are managed within the same PHCC [38]. All analyses will be performed on the individual level adjusted for clustering. Socioeconomic and demographic factors will be taken into consideration, by adjusting for these factors in the models. We have chosen mixed models because this method allows missing data on one of the two measurements. Primary analyses will include intention-to-treat analysis and per-protocol analysis.

#### Patient data

Cluster-adjusted regression models will be used for all kinds of outcomes, with both dichotomous and ordinal variables. Cluster-adjusted analysis of variance and linear regression will be used for continuous and normally distributed variables. Mann-Whitney tests will be used for nonparametric variables. The cluster-adjusted chi^2^ test (and cluster-adjusted logistic regression) will be used to compare proportions. Where appropriate, comparisons of arithmetic or geometric means will be performed. A possible substantively important imbalance arising on baseline variables will be controlled at the analysis stage, as adjustment for imbalanced variables. We intend to adjust for multiple comparisons, table-wise, by applying False Discovery Rate, based on the obtained *P* values.

#### Physician data

Analysis of the Physician Questionnaires will be performed using cluster-adjusted statistics. The emerging numeric data will be analysed using the cluster-adjusted chi^2^ test in order to compare proportions, and cluster-adjusted logistic regression for odds ratios [39]. Alternatively, if data is not normally distributed, nonparametric tests will be used.

*P* ≤ 0.05 will be considered statistically significant. For statistical analysis we will use software programmes STATA (StataCorp. 2015. Stata Statistical Software: Release 14; College Station, TX, USA: StataCorp LP.) and PSPP (IBM Corp. Released 2013. IBM SPSS Statistics for Windows, Version 22.0. Armonk, NY, USA: IBM Corp.).

## Discussion

COPD is considered a particularly complex clinical challenge involving managing diverse aspects of multimorbidity, symptom adaptation and lifestyle problematisation. The present study is expected to contribute scientific evidence for indicators of effective and feasible CME in COPD management aimed at PCPs. Hence, it would be of particular interest to study whether introducing advanced educational activities (SOLO 4–5) would lead to a more successful COPD care than basic education would alone (SOLO 1–3). More specifically, would a more interactive approach such as the Case Method be crucial for a behaviour change among PCPs so that their management of patients with COPD will improve?

### Patients

A limitation in the design is the lack of patients from the PHCCs in the reference group, i.e. a group of patients whose PCPs will not receive any CME intervention. It is a consequence of limited research resources. For other researches aiming at conducting similar studies we recommend ensuring that the financial resources are sufficient for an optimal design. Also, one of the main outcomes of the study is to evaluate two different educational methods, thus we find our setup of two arms sufficient to answer this research question. Another limitation is the introduction of possible bias due to patients changing care providers, i.e. PHCCs or PCPs during the study period, as the effect of our intervention becomes diluted in these cases. We plan to analyse the nonresponders to the second questionnaire, the patients who have changed PHCCs during the study time and the patients who otherwise become ineligible.

There are also pros and cons in analysing self-reported data through questionnaires instead of extracting data from national registers and medical records. Information, such as the number of exacerbations, hospitalisations and type of prescribed drugs, could be useful to collect through objective records as some patients may have problems with comprehending the facts, interpreting their symptoms in similar ways as health professionals do, or simply remembering this type of information retroactively. On the other hand, first-hand information on the patient’s current medication may increase the chances of forming the most accurate picture about a patient’s medication. Also, being able to collect data on the patients’ symptoms and quality of life is a substantial strength of this study.

Using an assumed ICC in the power calculation is always a limitation in a trial. However, we consider the chosen ICC of 0.01 as well motivated in the design. In the analysis, we will determine the observed ICC in our trial to see how well our assumed ICC predicted it.

### PCPs

The nature of the intervention, where the participants in arms 1 and 2 will be offered a CME whereas the reference group will not, may imply difficulties in recruiting reference participants. This affects our decision to recruit the reference group separately. The reason for the inclusion of a reference group is to assess a possible effect of general information on COPD that PHCs might receive as the national guidelines are to be revised during the study period. Also, despite mutual ILOs for the different types of CME, the CME leaders may use different presentation material at their respective CME sessions, which may introduce bias.

A limitation to our study is the use of a nonvalidated questionnaire for the PCPs. However, as our CME has introduced ILOs that have not been included in COPD training before, a validated method of measuring the effects in COPD management and treatment is not currently available. We have tried to enhance our chances of gathering satisfactory data by using diversity in design and analysis of data. The nonvalidated questionnaire together with the lack of previous studies mean that there is no primary endpoint measure to use for power calculation for PCPs. We plan to analyse the nonresponders to the first and second questionnaire.

### Planned reporting

The results will be published in scientific journals and presented in scientific conferences and other meetings.

### Trial status

The recruitment of PHCCs, PCPs and patients will be completed in 2016. Planned follow-ups will occur in 2016–2018.

## Additional file

Additional file 1: SPIRIT 2013 Checklist: recommended items to address in a clinical trial protocol and related documents*. (DOC 121 kb)
Additional file 2: Patient questionnaire. (DOC 132 kb)
Additional file 3: Case 1. (DOC 44 kb)
Additional file 4: Case 2. (DOC 44 mb)
Additional file 5: Physician Questionnaire. (DOC 124 kb)

## Abbreviations

CAT: The COPD Assessment Test
CCQ: The Clinical COPD Questionnaire
CME: Continuing medical education
CONSORT: Consolidated Standards of Reporting Trials
COPD: Chronic obstructive pulmonary disease
CRCT: Cluster randomised controlled trial
ICC: Intracluster correlation
ILO: Intended learning outcome
LINQ: The Lung Information Needs Questionnaire
M-rho: Matching correlation
PCP: Primary care physician
PHCC: Primary health care centre
RCT: Randomised controlled trial
S1–S5: SOLO levels 1–5
SOLO: Structure of Observed Learning Outcomes
SPIRIT: Standard Protocol Items: Recommendations for Interventional Trials

## References

[1] Stallberg B, Janson C, Johansson G, Larsson K, Stratelis G, Telg G (2014). Management, morbidity and mortality of COPD during an 11-year period: an observational retrospective epidemiological register study in Sweden (PATHOS). Prim Care Respir J.

[2] Global Initiative for Chronic Obstructive Lung Disease. 2013. http://www.goldcopd.com/. Accessed 1 May 2014.

[3] Lindberg A, Bjerg A, Ronmark E, Larsson LG, Lundback B (2006). Prevalence and underdiagnosis of COPD by disease severity and the attributable fraction of smoking Report from the Obstructive Lung Disease in Northern Sweden Studies. Respir Med.

[4] Lisspers K, Johansson G, Jansson C, Larsson K, Stratelis G, Hedegaard M (2014). Improvement in COPD management by access to asthma/COPD clinics in primary care: data from the observational PATHOS study. Respir Med.

[5] Pinnock H, Thomas M, Tsiligianni I, Lisspers K, Ostrem A, Stallberg B (2010). The International Primary Care Respiratory Group (IPCRG) Research Needs Statement. Prim Care Respir J.

[6] Lisspers K, Stallberg B, Hasselgren M, Johansson G, Svardsudd K (2009). Primary health care centres with asthma clinics: effects on patients knowledge and asthma control. Prim Care Respir J.

[7] Lloyd JS, Abrahamson S (1979). Effectiveness of continuing medical education: a review of the evidence. Eval Health Prof.

[8] Beaudry J (1989). The effectiveness of continuing medical education: a quantitative synthesis. J Contin Educ Health Prof.

[9] O’Brien MA, Rogers S, Jamtvedt G, Oxman AD, Odgaard-Jensen J, Kristoffersen DT (2007). Educational outreach visits: effects on professional practice and health care outcomes. Cochrane Database Syst Rev.

[10] Forsetlund L, Bjorndal A, Rashidian A, Jamtvedt G, O’Brien MA, Wolf F (2009). Continuing education meetings and workshops: effects on professional practice and health care outcomes. Cochrane Database Syst Rev.

[11] Nordquist J (2009). Att undervisa med case i utbildningar inom hälso- och sjukvården.

[12] Kiessling A, Lewitt M, Henriksson P (2011). Case-based training of evidence-based clinical practice in primary care and decreased mortality in patients with coronary heart disease. Ann Fam Med.

[13] Drexel C, Jacobson A, Hanania NA, Whitfield B, Katz J, Sullivan T (2011). Measuring the impact of a live, case-based, multiformat, interactive continuing medical education program on improving clinician knowledge and competency in evidence-based COPD care. Int J Chron Obstruct Pulmon Dis.

[14] van der Molen T, Willemse BW, Schokker S, ten Hacken NH, Postma DS, Juniper EF (2003). Development, validity and responsiveness of the Clinical COPD Questionnaire. Health Qual Life Outcomes.

[15] Jones PW, Harding G, Berry P, Wiklund I, Chen WH, Kline LN (2009). Development and first validation of the COPD Assessment Test. Eur Respir J.

[16] Jones RC, Wang X, Harding S, Bott J, Hyland M (2008). Educational impact of pulmonary rehabilitation: lung information needs questionnaire. Respir Med.

[17] Chan AW, Tetzlaff JM, Gotzsche PC, Altman DG, Mann H, Berlin JA (2013). SPIRIT 2013 explanation and elaboration: guidance for protocols of clinical trials. BMJ.

[18] Campbell MK, Piaggio G, Elbourne DR, Altman DG (2012). Consort 2010 statement: extension to cluster randomised trials. BMJ.

[19] Mauffette-Leenders LA, Erskine JA, Leenders MR (1997). Learning with cases.

[20] Bologna Declaration. Helsinki: European Association for Quality Assurance in Higher Education. 2005. http://www.ond.vlaanderen.be/hogeronderwijs/bologna/documents/Standards-and-Guidelines-for-QA.pdf. . Accessed 1 May 2014.

[21] Biggs J, Tang C, editors. Teaching for quality learning at university: The McGraw-Hill Companies. Maidenhead: Biggs; 2011.

[22] Biggs JB, Collis KF (1982). Evaluating the quality of learning: the SOLO taxonomy (structure of the observed learning outcome).

[23] Govaerts MJB, Schuwirth LWT, Van der Vleuten CPM, Muijtjens AMM (2011). Workplace-based assessment: effects of rater expertise. Adv Health Sci Educ.

[24] Svensk Lungmedicinsk Förening. Nationellt vårdprogram för KOL, Kroniskt Obstruktiv Lungsjukdom. http://www.slmf.se/KOL/ 2013. Accessed 1 May 2014.

[25] Bylin G, Hansson B, Larsson S, Meyer P, Ställberg B, Sundberg R. Socialstyrelsens riktlinjer för vård av astma och kroniskt obstruktiv lungsjukdom (KOL). 2004. Socialstyrelsen.

[26] Läkemedelsverket. Läkemedelsbehandling av kroniskt obstruktiv lungsjukdom (KOL). Socialstyrelsen, Sverige; 2009. https://lakemedelsverket.se/upload/halso-ochsjukvard/behandlingsrekommendationer/KOL_rek_webb_bokm.pdf. Accessed 1 May 2014.

[27] Sandelowsky H, Hylander I, Krakau I, Modin S, Stallberg B, Nager A (2016). Time pressured deprioritization of COPD in primary care: a qualitative study. Scand J Prim Health Care.

[28] Kocks JW, Tuinenga MG, Uil SM, van den Berg JW, Stahl E, van der Molen T (2006). Health status measurement in COPD: the minimal clinically important difference of the clinical COPD questionnaire. Respir Res.

[29] van Boven JF, Stuurman-Bieze AG, Hiddink EG, Postma MJ (2016). Effects of targeting disease and medication management interventions towards patients with COPD. Curr Med Res Opin.

[30] Trappenburg JC, Monninkhof EM, Bourbeau J, Troosters T, Schrijvers AJ, Verheij TJ (2011). Effect of an action plan with ongoing support by a case manager on exacerbation-related outcome in patients with COPD: a multicentre randomised controlled trial. Thorax.

[31] Kon SS, Canavan JL, Jones SE, Nolan CM, Clark AL, Dickson MJ (2014). Minimum clinically important difference for the COPD Assessment Test: a prospective analysis. Lancet Respir Med.

[32] Jones R. LINQ Scoring Template. 2011. In: http://www.linq.org.uk/PDFscoring/LINQscoringtool.pdf. University of Plymouth, School of Psychology. Accessed 1 May 2014.

[33] Adams G, Gulliford MC, Ukoumunne OC, Eldridge S, Chinn S, Campbell MJ (2004). Patterns of intra-cluster correlation from primary care research to inform study design and analysis. J Clin Epidemiol.

[34] Killip S, Mahfoud Z, Pearce K (2004). What is an intracluster correlation coefficient? Crucial concepts for primary care researchers. Ann Fam Med.

[35] Hayes RJMH (2009). Cluster randomised trials.

[36] Pyke SD, Wood DA, Kinmonth AL, Thompson SG (1997). Change in coronary risk and coronary risk factor levels in couples following lifestyle intervention. The British Family Heart Study. Arch Fam Med.

[37] Martin DC, Diehr P, Perrin EB, Koepsell TD (1993). The effect of matching on the power of randomized community intervention studies. Stat Med.

[38] Campbell MK, Mollison J, Steen N, Grimshaw JM, Eccles M (2000). Analysis of cluster randomized trials in primary care: a practical approach. Fam Pract.

[39] Reed JF (2004). Adjusted chi-square statistics: application to clustered binary data in primary care. Ann Fam Med.

