# Supplementary material for: Case Method in COPD education for primary care physicians: study protocol for a cluster randomised controlled trial
Source: Trials. 2017 Apr 27;18:197. doi: 10.1186/s13063-017-1889-4 (PMC5408477; doi:10.1186/s13063-017-1889-4)
Supplement: Additional file 2: — Patient questionnaire. (DOC 132 kb) [file 13063_2017_1889_MOESM2_ESM.doc]

## ***Questions about your lung disease***

Additional file 2. Patient questionnaire.

| **1.** | **How old are you?** …..…..….. |
| --- | --- |
| **2.** | **At which health clinic/medical centre/doctor’s practice is your doctor based?**    ……………………………………………………………….  Not applicable |
| **3.** | **What lung disease do you have? Tick whichever option(s) you think apply.**  COPD  Asthma  Chronic bronchitis  Other:____________________  Don’t know  I have never had respirator problems  I have no lung disease |
| **4.** | **How old were you when your lung disease started to trouble you?**  Under 30  30-50  51-60  61-70  Over 70 |
| **5.** | Do you have/have you ever had the following diseases?(Tick the appropriate box(es)). Diabetes  Heart disease  Stroke  Hypertension  Anxiety/Depression  Sleep apnoea  Rheumatic disease  Cancer  Heartburn  Chronic pain  None of these |
| **6.** | **Have you ever been medically diagnosed with asthma?**  Yes  No  Don’t know |

|  | *Questions about your medication* |
| --- | --- |
| **7.** | **Have you taken fast-acting bronchial dilator such as Bricanyl, Ventoline, Airomir, Airsalb, Salbutamol, Ventilastin or Buventol in the *past week* for respiratory problems?**  Yes   No  Don’t know |
| **8.** | **Have you used a cortisone inhalant such as Pulmicort, Flutide, Becotide, Asmanex, Giona, Novopulmon, Budesonid, Aerobec, Alvesco or Beclomet in the *past six months*?**  Regularly  Occasionally  No   Don’t know |
| **9.** | **Have you used the bronchial dilator Atrovent in the *past six months*?**  Regularly  Occasionally  Once or twice  No  Don’t know |
| **10.** | **Have you used any of the bronchial dilators Spiriva, Eklira or Seebri in the *past six months*?**  Regularly  Occasionally  No  Don’t know |
| **11.** | **Have you used the long-acting bronchial dilators such as Oxis, Serevent, Formatris or Onbrez in the *past six months*?**  Regularly  Occasionally  No  Don’t know |
| **12.** | **Have you taken *extra doses* of long-acting bronchial dilators such as Oxis, Serevent, Formatris or Onbrez in the *past week*?**  Yes  No  Don’t know |
| **13.** | **Have you used Symbicort, Seretide, Airflusal, Relanio, Bufomix, Flutiform or Innovair (combination of long-acting bronchial dilator and cortisone) in the *past six months*?**  Regularly  Occasionally  No  Don’t know |
| **14.** | **Have you taken *extra doses* of Symbicort, Seretide, Airflusal, Relanio, Bufomix, Flutiform or Innovair in the past week?**  Yes  No  Don’t know |
| **15.** | **Have you used Daxas in the *past six months*?**  Regularly  Occasionally  No  Don’t know |
| **16.** | **Have you needed to take *cortisone tablets* (Betapred or Prednisolon) on account of a deterioration in your lung disease in the *past six months*?**  Yes, once  Yes, on two separate occasions  Yes, on more than two separate occasions  I take cortisone tablets regularly  No |
| **17.** | **Have you needed to take *antibiotics* on account of a deterioration in your lung disease at any time in the *past six months*?**  Yes, once  Yes, on two separate occasions  Yes, on more than two separate occasions  No |
| **18.** | **Bearing in mind how you use your respiratory medication and what your doctor or asthma/COPD nurse has recommended, which statement applies the closest to you?**  I always take what’s recommended  I usually take what’s recommended  I sometimes take what’s recommended  I rarely take what’s recommended  I never take what’s recommended  I don’t take respiratory medication |
| **19.** | **Have you been prescribed medicine for preventing or treating osteoporosis in *the past year*?**  Yes  No  Don’t know |

|  | *Questions about the problems your lung disease causes you* |
| --- | --- |
| **20.** | **Have you been woken at night by coughing, wheezing or respiratory difficulties in *the past week*?**  Yes, once  Yes, several times  No |
| **21.** | **Have you made an emergency appointment with your doctor/medical centre on account of a deterioration in your lung disease in the *past six months*?**  Yes, once  Yes, twice  Yes, more than twice  No |
| **22.** | **Have you sought emergency help from a hospital on account of a deterioration in your lung disease in the *past six months*?**  Yes, once  Yes, twice  Yes, more than twice  No |
| **23.** | **Have you been in hospital on account of your lung disease in the *past six months*?**  Yes, once  Yes, twice  Yes, more than twice  No |
| **24.** | Have you sought any kind of emergency medical help on account of a deterioration in your lung disease in t*he past year*?  Yes, once  Yes, twice  Yes, three times  Yes, more than three times  No |
| **25.** | **How would you describe the severity of your lung disease?**  Very mild  Mild  Moderately severe  Severe  Very severe  I have no lung disease |
| **26.** | **How often and when do you suffer from breathlessness? Tick whichever option(s) you think apply:**  When I really exert myself, not when I go for a quick walk or walk uphill.  When I go for a quick walk or walk uphill.  When I walk on level ground at the same pace as another person of my own age.  I get so breathless when I walk on level ground that I have to stop despite going at my own pace.  When I wash or dress myself. |

|  | ***Questions about your dealings with the medical services*** |
| --- | --- |
| **27.** | **Where do you normally go to have your lung disease checked? Tick the box(es):**  My local GP  My occupational physician  The hospital (pulmonary or medical clinic)  A private pulmonologist  An asthma/COPD nurse  Other  Nowhere  I have no lung disease (go to question 42) |
| **28.** | **Do you know which doctor is responsible for treating your lung disease?**  Yes   No |
| **29.** | Have you visited an asthma/COPD nurse on account of your lung disease in the past year? (NOT emergency visits)  Yes  No  Don’t know |
| **30.** | Have you been to see a physiotherapist on account of your lung disease in the past year? Yes  No  Don’t know |
| **31.** | Have you been to see an occupational therapist on account of your lung disease in the past year? Yes  No  Don’t know |
| **32.** | Have you been to see a dietician on account of your lung disease in the past year? Yes  No  Don’t know |
| **33.** | Have you been to see a counsellor on account of your lung disease in the past year? Yes  No  Don’t know |
| **34.** | Do you think you are suitably informed about how to handle a deterioration in your lung disease? Yes  Yes, somewhat  Yes, a little  No |
| **35.** | **Have you been given a pneumococcal vaccine in the past 5 years?**  Yes  No  Don’t know |
| **36.** | **Have you been given an influenza vaccine in the past 12 months?**  Yes  No  Don’t know |

|  | | ***Other questions*** |
| --- | --- | --- |
| **37.** | | **What is the *highest* level of education you have achieved?**  Less than 5 years in school  Primary school  Grammar school or the equivalent  2-year upper secondary/vocational college  3/4-year upper secondary  University or university college, up to 2.5 years  University or university college, 3 years or more |
| **38.** | | **How tall are you?** ……………… cm |
| **39.** | | **How much do you weigh?** ……………kg |
| **40.** | **If you smoke/have smoked: for how many years have you been/were you a daily smoker?** ………… | |
| **41.** | **If you smoke/have smoked: How many cigarettes do/did you smoke a day on average?**......................... | |
| **42.** | **Answer this question if you smoke or stopped smoking in the past 5 years.**  **Have you *been* *offered* professional medical help to quit smoking?**  Yes  No  Don’t know | |
| **43.** | **Answer this question if you smoke or stopped smoking in the past 5 years.**  **Have you *been* *given* professional medical help to quit smoking, either individually or in a group?**  Yes  No  Don’t know | |
| **44.** | **Answer this question if you smoke/have smoked.**  **Have you used drugs, OTC or prescription, in order to quit smoking?**  Yes, nicotine replacement (e.g. patches or chewing gum)  Pills to reduce craving (Zyban or Champix)  Other ……………………………………………. No | |

***There is a final set of eight questions on the next page***

(COPD Assessment Test = CAT. Reference http://www.catestonline.org)
