# Supplementary material for: Case Method in COPD education for primary care physicians: study protocol for a cluster randomised controlled trial
Source: Trials. 2017 Apr 27;18:197. doi: 10.1186/s13063-017-1889-4 (PMC5408477; doi:10.1186/s13063-017-1889-4)
Supplement: Additional file 3: — Case 1. (DOC 44 kb) [file 13063_2017_1889_MOESM3_ESM.doc]

The Super Doctor

Additional file 3. Case 1.

District family physician Karin Adamsson had recently returned from maternity leave and was keen to get back to work. It was a sunny day in October and she was scheduled to be on duty as emergency doctor at her health care centre that morning. Emergency shifts were understandably stressful, but today was a good day. There were always fewer patients in the middle of the week, the lull before the weekend storm. Karin had managed to drop her two-year-old off at preschool without too much fuss, and was in her office, sipping coffee as she fired up her computer in readiness to take on her patients for the day. There would be at least ten, sometimes as many as twelve, of a morning. Ten to fifteen minute consultations with patients with some or other complaint – she would get through them swiftly and efficiently. “Time to produce some care”, she thought a little cynically. The system was what it was these days, and you just had to grin and bear it, despite the stress. But then again, she had to admit that she enjoyed the praise she received for being so efficient. She was hoping for lots of urinary tract infections or tonsillitis, because she could deal with them quickly.

Karin’s day in her surgery started off as expected, and the first three patients came and went. She had just received her fourth, a woman in her 40s with a cough, when the emergency sister Maria popped her head round the door.

“Hey, Karin, would you be able to come down when you’re done with this patient, I’ve got someone with respiratory problems out there. It’s a regular. I need a prescription for the inhalations.”

“Er, OK,” was all Karin could manage before Maria’s head disappeared from the doorway.

With her stethoscope hanging round her neck, Karin exchanged a few words with her patient, urging her to think about her smoking habit and welcoming her back if she didn’t feel better. A cough medicine prescription then shot off into cyberspace and Karin into the corridor.

She found a middle-aged man sitting alone in the emergency room with the door ajar. Karin briefly introduced herself to the ashen, slender figure sitting slightly hunched on the chair, smelling of smoke and with his arms hanging by his sides.

“Kalle,” said the man.

He was taciturn and short of breath, with an intense look in his eyes. Karin had never seen him before. “*A regular, Maria said*,” she thought. “*I suppose I’ll just have to get busy with the inhalation.* On the paper sheet of his stretcher, Maria had written in pen:

“Lennart Karlberg, 490612-xxxx, O2sat 89%, PEF 200, B/P 150/90, P 90, temp 36.8.”

Karin listened to Kalle’s lungs and established that his condition was obstructive. She called in Maria, who started administering the prescribed inhalations.

Back in her office, a quick look at Kalle’s drug list told her that Kalle was a heavy consumer of Doxyferm and cough medicines, and had had a number of prescriptions for Predisolon. The diagnosis list consisted of a single diagnosis – COPD – listed 10 times. “*It doesn’t say that he was a smoker, but you could tell by the smell”,* thought Karin as she noted that Kalle was registered to one of her colleagues, who was currently on holiday.

Twenty minutes later, Maria reappeared at her door. Karin was just finishing off lancing a boil on a 20-year-old patient – yes, in her own office, since the operating room was occupied by a colleague.

“He’s done,” said Maria quickly and rushed out again.

Karin asked the nursing auxiliary to dress the boil and checked her appointments list: five patients left. It was 10.45 and she had missed the morning coffee break. “*Which is just as well, as I wouldn’t have had time to go to the loo afterwards,*” she thought sarcastically to herself, and returned to the emergency room to check up on Kalle.

Kalle was breathing more easily now. His lungs sounded much clearer; his saturation had climbed to 95% and his PEF to 220. He was a little more talkative now and his gaze less intense. He gave a little smile and drawled:

“I was out with my mates for a beer yesterday, but then last night found it a real bugger to breathe. Got an ear-bashing from my grandkids who came calling the other say, didn’t I, the snotty lot. So I like to come here to breathe in a little medicine now and then. Reckoned I’d come tomorrow too. I normally do that. Damn good stuff this medicine. Smoked? Hell, yeah, for at least 50 years! But not these days, no way.”

Karin regarded Maria’s “regular”. *“What a charmer”,* she thought, although knowing too that his COPD was beyond repair. He should stop smoking, take the right medicine…and all that. She knew the treatment ladder for COPD, but felt it best to look it up anyway as she was a little rusty after having been away. But she was not really convinced it was worth the bother: Kalle seemed to be a man of dubious lifestyle, and given that his COPD was self-inflicted he should seek help for it himself. And besides, there were five patients still in the waiting room, all of them coughing and sniffling. And someone might even come in with a serious wound to dress or chest pains. Karin was starting to get hungry too – would she get a break for lunch? No, there was no time for chatting with Kalle about stopping smoking or adjusting his medicine. “*Got to keep up the pace. Emergencies are emergencies, that’s my concern for today.”*

She prescribed a five-day course of cortisone and antibiotics, renewed Kalle’s prescription for Bricanyl, and said to him:

“Come back if you don’t feel any better.”
