# Supplementary material for: Case Method in COPD education for primary care physicians: study protocol for a cluster randomised controlled trial
Source: Trials. 2017 Apr 27;18:197. doi: 10.1186/s13063-017-1889-4 (PMC5408477; doi:10.1186/s13063-017-1889-4)
Supplement: Additional file 4: — Case 2. (DOC 44 mb) [file 13063_2017_1889_MOESM4_ESM.doc]

Additional file 4. Case 2.

**The ingrained smell of smoke**

**2007**

Lars Holmgren was a resident physician in a medium-sized primary health care centre in a small town on the coast, 10 miles or so away from the local hospital. He had lived with his wife and two children in this town for his entire adult life. Lars was a keen sportsman in his 30s, and known locally as the skilled, cheerful and pleasant doctor. He was involved in his children’s own sports and since he was interested in lifestyle issues, he also worked with an anti-smoking campaign for teenagers. He was a non-smoker, and always had been.

It was a normal day in November. Lars could feel the cold and damp from the sea working its way into the town as he walked the short distance from the restaurant where he had had his lunch back to the clinic. Stepping into the waiting room, he spotted a familiar face: Gerd Alvén, a cashier from his local supermarket. A few years back he had taken care of Gerd’s husband’s – Gösta’s – COPD, and she had always accompanied him to his appointments. Gösta had recently died, apparently of a heart attack. Lars ushered Gerd into his surgery, and as she waddled into the room she was pursued by the familiar rank smell of stale tobacco. She panted slightly as she perched herself on the chair. She was a little overweight, and looked older than her 62 years. Lars asked her how he could help.

“I’ve got such a pain in my back… I’ve had it for ages, but now it’s got so bad I can’t sleep at night, can’t get myself into a good position. And my legs are playing up too, it’s the knee you know, it gives me grief when I walk. Ugh, I just feel so old and tired.”

*“Old, tired, pain in the back and legs. She’s not that old… But she’s been smoking for years, I wonder if she’s on the bottle too? Slightly overweight, the knees are taking a hammering…”,* Lars tried to sum up the situation. He knew little about Gerd, and seeing her now called to mind her deceased husband and he felt a pang of guilt about her. She probably also had COPD as well. He had noticed that she had been slightly out of breath when visiting with Gösta… Oh, yes, Lars knew Gerd had her own troubles alright, it was just that when he had met her before the focus had always been on Gösta. He had been the sick one! She had never mentioned herself at these meetings. Gösta had been the important one, her lifelong companion, her everything. She had been terrified that Gösta would soon be taken from her. Lars felt that Gerd had never been interested in talking about her own troubles.

And then suddenly she said:

“And then there’s this blasted cough of mine, too.”

“No, I’ve not had a cold particularly. It’s just this cough…and the back. I find it hard to sleep at times. I’ve not had a proper night’s sleep since…since…my husband passed away. I feel so lonely. I miss him so much. I don’t really know what to do with myself when I get home from work.”

Lars established that neither her back nor her knee showed any sign of disease or injury and her lungs sounded fine, with possibly only a little faint respiratory sound. Lars thought, again, about CODP. When he suggested a spirometric examination, she willingly agreed, but clearly became anxious and nervy. She understood, of course, that she might also be diagnosed with this fatal disease. But she wanted to do as the doctor suggested.

A few weeks later, Lars saw her spirometry results.

Gerd had FEV1/FVC 0.40 and FEV1 at 48% of expected value.

Lars called her back and informed her that she had COPD. He prescribed medicine for her and they had the following exchange:

“Listen, Gerd, you have to stop smoking. This doesn’t look good, you know.”

“I know. I know all that,” she replied crossly.

“So what’s making you carry on?”

“I tried quitting before, but it didn’t work. I can’t give up. And I don’t want to, either.”

Even though Lars thought it difficult to discuss quitting with Gerd, he did manage to make her see Sister Eva. Eva was considered a skilled asthma/COPD nurse – she was even known as the “stop-smoking sister” – and was adept at conducting and interpreting spirometry tests. However, despite Eva’s brave attempts to motivate Gerd, she simply was uninterested in giving up as, by her own account, she had nothing else to do in the evenings, and smoking eased her nerves.

“I don’t want to talk about that, I don’t want to discuss it!” exclaimed Gerd.

They agreed that Gerd would get in touch if ever she changed her mind.

**2010**

Lars had recently become a registered general practitioner, and despite the considerable increase in his workload he still enjoyed his job. Gerd was becoming a regular at the clinic’s new drop-in centre. He had also received a few follow-up referrals from the hospital, where Gerd had sought emergency help for respiratory problems. On her revisits to Lars, she usually said that she felt “no different” and wanted no more help.

One hospital referral read: *“Diagnosis: Pneumonia, COPD and heart failure. Requested examination: Grateful for follow-up and EL status within a week”.*  Lars reacted to the heart-failure diagnosis and to the fact that Gerd had been put on metoprolol and a diuretic.

On her next revisit, Lars asked Gerd about her time at the hospital.

“The hospital was great! I was so poorly, you know… But the girls on the ward were so kind. I got better, they gave me penicillin and cortisone, you know, and I had to inhale medicine several times a day. But…it’s not nice lying in the same room as lots of other people, people who were even worse off than me. And I hated the food. Ugh! And then I saw a different doctor every day, although they were all nice to me too. I was there for three days. But you know, I haven’t managed a THING since coming home. Lucky that my neighbour’s so helpful.”

Gerd started to have difficulty going to the supermarket despite living only a couple of hundred yards away, and she realised that it was her knee that was at fault. She also grew very short of breath. It was all too clear that she was far too sick to work. Lars contacted the Social Insurance Agency and suggested to Gerd that she have a talk with Sister Eva again. This time she relented.

When she saw Eva, Gerd still found it hard to talk about smoking, although she did consent to let her help her with this and that: she sorted out all the different medicines that Gerd had had over the years and suggested that she use a rollator.

“It’s the best thing that’s ever happened to me!” exclaimed Gerd after having received her new rollator.

Epilogue (To be read aloud by the educator to the participants at the end)

Gerd started to feel that she needed to see Eva and Lars more and more often as her exacerbations grew gradually worse. Her habitual state seemed also to have become increasingly breathless. Lars thought she had started to lose weight too, which was confirmed by Eva, who regularly weighed her – something that Lars had introduced as routine for all their COPD patients. Unfortunately, Gerd had not quit smoking.

Lars updated her medication and Gerd showed no interest in a quit-smoking programme or physiotherapy. She occasionally ended up in hospital again, but did not like it there and went to see or called Lars and Eva instead. Sometimes they managed her treatment completely during one exacerbation period, allowing her to attend the clinic several days in a row for her inhalations. Usually her saturation was at 91-92%, a little lower during exacerbations. Gerd was spared the trouble of having to go to hospital.

But Gerd remained anxious and tired, and eventually refused to socialise. Her COPD had developed to stage 4. COPD was a constant presence: she found it hard to laugh, to cry – even to eat. It quite simply made her too short of breath and tired.

Since she was a smoker, she could not be given oxygen despite her respiratory failure. Lars adjusted her medication and started to focus all the more on her depressive tendency. Gerd was prescribed an SSRI which she dutifully took, and had brief contact with a counsellor. She preferred, however, to speak to Lars and Eva. Her visits could be short, sometimes 10 minutes, and it became obvious that she just needed to talk. Her conversation often turned to the themes of death and helplessness. With Lars and Eva she felt safe.

At the age of 72, six years after her diagnosis, she was ravaged by COPD. One day she did not wake up. She had died at home, alone.

*“Poor Gerd. If only she’d stopped smoking. If only we could have made her quit…”* Lars wondered privately if there was anything he could have done differently.
