# Supplementary material for: Case Method in COPD education for primary care physicians: study protocol for a cluster randomised controlled trial
Source: Trials. 2017 Apr 27;18:197. doi: 10.1186/s13063-017-1889-4 (PMC5408477; doi:10.1186/s13063-017-1889-4)
Supplement: Additional file 5: — Physician Questionnaire. (DOC 124 kb) [file 13063_2017_1889_MOESM5_ESM.doc]

| **Case I**  *Your patient, a 59-year old male computer technician, comes to see you complaining of severe breathlessness in the past year, especially when exerting himself. He has attributed it to being unfit, since he doesn’t like exercising. He has smoked for all his adult life. He also has a phlegmy morning cough. He has no known allergies and no pets. His children are worried about him, hence his appointment with you. He says his family go on at him about his smoking, but he has no intention of quitting.* | | |  |
| --- | --- | --- | --- |
| Question 1 | After a clinical examination, you suspect him of having COPD. Which of the following options (you may choose more than one answer) form part of your initial investigation of this patient? | |  |
|  | a | PEF measurement | |
|  | b | Spirometry | |
|  | c | Lung X-ray | |
|  | d | PEF curve | |
|  | e | ProBNP | |
|  | f | BMI | |
| *His spirometry reading:* | | |  |

|  | | Before bronchial dilation | | After bronchial dilation | |  |
| --- | --- | --- | --- | --- | --- | --- |
| Variable | Normal value | Recorded value | % of normal | Recorded value | % of normal | % change |
| VC | 5.2 | 3.5 | 67 | 3.6 | 69 | +3 |
| FVC | 5.2 | 3.0 | 58 | 3.0 | 58 | +3 |
| FEV1 | 3.9 | 1.7 | 44 | 2.0 | 56 | +18 |
| FEV1/VC | 0.75 | 0.49 | 65 | 0.55 | 73 | +12 |
| FEV1/FVC | 0.75 | 0.57 | 76 | 0.67 | 89 | +18 |

| Question 2 | Going by his spirometry values, the most probable diagnosis is: | |
| --- | --- | --- |
|  | a | Asthma |
|  | b | COPD, stage 2 |
|  | c | Asthma and COPD, stage 2 |
|  | d | COPD, stage 1 |
|  | e | Asthma and COPD, stage 3 |
|  | f | COPD, stage 3 |
|  | g | Neither asthma nor COPD |
| Question 3 | How do you deal with his reluctance to quit smoking? | |
|  | Write your answer here | |
|  |  | |
|  |  | |
| **Case II**  *You are the emergency doctor on duty today at your medical centre. A 59-year old female smoker arrives complaining of an increase in breathlessness, phlegm and expectoration over the past few days.*  *You see from her records that she has been registered to your colleague, who ordered a spirometric examination four years ago on account of the patient being a smoker and having a cough for which she sought medical attention. Spirometry revealed COPD with an FEV1 reading at 60% of the expected value.*  *You also see that she had been prescribed an expectorant, a fast-acting beta-2 stimulant in dry powder inhalator form (with some repeat prescriptions) and antibiotics for a urinary infection.*  *You can hear that she is breathless and obstructive and that she has a cold. She has a temperature of 37.1 degrees and a CRP of 26, saturation 91%.*  *You send her for bronchial dilatory inhalations. She subsequently feels better and her saturation rises to 95 %.* | | |
| Question 4 | With which of the following drugs do you choose to treat her current symptoms following the emergency bronchial dilation (you may choose more than one answer)? | |
|  | a | One dose of Betapred, 16 tablets |
|  | b | One dose Betapred, 8 tablets |
|  | c | Doxycyklin (oral) |
|  | d | Amoxicillin (oral) |
|  | e | PcV |
|  | f | Steroids, e.g. prednisolon 20-30 mg per day, orally for 5-10 days |
|  | g | Steroids, e.g. prednisolon 5-10 mg per day, orally for 5-10 days |
|  | h | Inhaled steroids, high dose for 14 days |
| Question 5 | Do you feel this patient needs monitoring? If yes, how? If no, why not? | |
|  | Write your answer here | |
|  |  |  |
|  |  |  |
|  |  |  |
| Question 6 | She wants to quit smoking and asks for your help. What smoking cessation method do you recommend? | |
|  | Write your answer here | |
|  |  | |
|  |  | |
|  |  | |

| **Case III**  *You meet a 60-year old male patient with previously untreated COPD. Spirometry shows FEV1 at 71% of the expected value. The man quit smoking a couple of years ago and he has no medical history of acute exacerbation periods. He now experiences increasing breathlessness while out walking, gardening and doing other effortful activities.* | | |
| --- | --- | --- |
| Question 7 | Which of the following treatment options would you recommend for this patient if you wish to start maintenance therapy (you may choose more than one answer)? | |
|  | a | Only short-acting beta 2 stimulants p.r.n. |
|  | b | Long-acting beta-2 stimulants |
|  | c | Short-acting anticholinergics |
|  | d | Long-acting anticholinergics |
|  | e | A combination of long-acting beta-2 stimulants and long-acting anticholinergics |
|  | f | Inhaled steroids |
|  | g | A combination preparation of long-acting beta-2 stimulants and inhaled steroids (e.g. Symbicort Forte® or Seretide Forte®) |
|  | h | Roflumilast (Daxas®) |
|  | I | Acetylcysteine effervescent tablets |
| Question 8 | The patient also has heart failure, which is common in patients with COPD. When it comes to treating it with beta blockers in a “normal case”, which of the following actions are **wrong** (you may choose more than one answer)? | |
|  | a | To opt for a beta-1 selective beta blocker, such as karvedidol (Kredex®) |
|  | b | To opt for a beta blocker as per the heart failure recommendations, e.g. metoprolol |
|  | c | To opt for no beta blockers |
|  | d | To opt for a beta blocker as in (b) and to increase the beta agonist (beta-2 stimulant) in inhaled form |
| Question 9 | When and how do you monitor the patient after your administration of maintenance therapy for COPD? | |
|  | Write your answer here | |
|  |  |  |
|  |  |  |
|  |  |  |
|  |  |  |

| **Case IV**  *Your patient is a 65-year old female ex-smoker with stage 3 COPD. She has had a difficult year with three long exacerbation periods with obsructivity, and has repeatedly received treatment from a hospital emergency unit and her local medical centre. In the past few years she has met the centre’s COPD nurse every six months or so. During a follow-up appointment, you find that she feels fine but has lost weight, and loses her breath a little during conversation, especially directly after having walked some way down the corridor. Her saturation value is currently 93%, and she has a BMI of 21.* | | |
| --- | --- | --- |
| Question 10 | What do you do (you may choose more than one answer)? | |
|  | a | Refer her to the lung clinic |
|  | b | Refer her to the physiotherapist |
|  | c | Advise her to eat an extra energy-rich diet |
|  | d | Refer her to a district nurse/dietician for a dietary consultation and prescribe a liquid nutritional supplement |
|  | e | Prescribe FYSS (Physical Activity in the Prevention and Treatment of Disease) |
|  | f | Send her for an arterial blood gas test |
|  | g | Check that she is on optimal medication |
| Question 11 | **In which of the following situations would it be most appropriate for you to suspect respiratory insufficiency and send the patient for an arterial blood gas test?** | |
|  | a | The patient has started to experience exacerbations |
|  | b | **The patient’s FEV1 is < 40% of the expected value** |
|  | c | **The patient’s saturation at rest is < 92%** |
|  | d | **The patient’s saturation drops to** < 90 % on exertion |
|  | e | **The patient’s saturation drops to** < 92% on exertion |
|  | f | **The patient’s saturation at rest is < 94%** |
|  | g | The patient feels the drugs are not helping |

| **Case V**  *A 70-year old male smoker with heart failure, hypertension, COPD, mild depression and chronic back pain came to see you a year ago. He is taking Spiriva®, Enalapril, Lasix Retard®, Metoprolol, Citalopram and Alvedon®, and has now come for his annual checkup. He seems to be in good health. You open the conversation by asking how he is.*  Two possible scenarios now present themselves (A and B):   1. *The patient says he’s fine, but he mainly wants to have a PSA test, to renew his prescriptions and to get help with his bad back.* | |
| --- | --- |
| Question 12 | How do you deal with the patient? |
|  | Write your answer here |
|  |
|  |
|  |
|  |
| 1. *The patient says he has no energy and gets easily out of breath.* | |
| Question 13 | How do you deal with the patient? |
|  | Write your answer here |
|  |
|  |
|  |
|  |
